# Modern Management of Fournier’s Gangrene

**DOI:** 10.1007/s11934-025-01275-3

**Published:** 2025-06-02

**Authors:** Kyle J. Kopechek, Hiren V. Patel, George E. Koch

**Affiliations:** 1 Department of Urology, The Ohio State University Wexner Medical Center, 915 Olentangy River Road, Suite 3117, Columbus, OH 43212 USA; 2 Department of Urology, The University of Washington, Seattle, WA USA

**Keywords:** Fournier’s gangrene, Necrotizing soft tissue infection, Skin-sparing debridement, Wound care, Genital reconstruction, Wound closure

## Abstract

**Purpose of review:**

This review explores new evidence in Fournier’s Gangrene management, emphasizing survivorship. We highlight the shift toward skin-sparing debridement techniques, new reconstructive strategies, and highlight limited evidence on outcomes. Additionally, we examine recent evidence on diagnosis, antimicrobial therapy, adjunctive treatments, and post-operative wound care.

**Recent findings:**

New evidence supports the feasibility of skin-sparing debridement, reducing the need for extensive reconstruction while improving primary closure rates and lowering healthcare costs. Advances in reconstructive techniques accelerate wound healing and shorten hospital stays. Optimized wound management—integrating antimicrobial solutions, negative pressure therapy, and targeted antibiotics—continues to improve recovery while minimizing morbidity and mortality.

**Summary:**

Modern Fournier’s management prioritizes early recognition, tissue preservation, and early genital reconstruction. Despite advancements, gaps remain in early diagnosis and long-term outcomes after the index admission. Further research on post-reconstruction recovery is essential to refine treatment protocols and determine quality of life for affected patients.

## Introduction

Fournier’s Gangrene (FG) is a life-threatening necrotizing soft tissue infection (NSTI) characterized by rapid tissue destruction along fascial planes, primarily affecting the perineum, genitalia, and lower abdominal wall. This devastating condition arises from a polymicrobial infection and propagates due to thrombosis of subcutaneous vessels, subsequent tissue ischemia, superinfection and necrosis. FG presents from diverse origins and clinical manifestations, including cutaneous injury or infection and violation of the gastrointestinal or genitourinary tracts. While prompt diagnosis, immediate initiation of broad-spectrum antibiotics, aggressive management of comorbidities, and urgent surgical debridement remain the cornerstones of treatment, the contemporary surgical approach is evolving. There is a growing emphasis on a reconstructive-focused, patient-centered strategy incorporating skin-sparing debridement and a variety of advanced reconstructive techniques to optimize functional outcomes. As critical care medicine continues to lower the mortality rate of FG, an emphasis on survivorship and returning the patient to a normal life are quickly becoming the standard of care.

## Epidemiology, Risk Factors, and Mortality

### Epidemiology

FG accounts for approximately 0.02% of annual hospital admissions and has an incidence of 1.6 per 100,000 males, predominantly affecting those between 50 and 79 years of age [1]. While historically FG was thought to display a strong male predominance, more recent evidence suggests females make up 20–30% of cases [2, 3]. In fact, females are more likely to die from FG with mortality rates of 7.1% vs. 5.7% in males, with non-white females experiencing worse outcomes [4]. FG remains a serious risk for both genders, underscoring the importance of recognizing diverse presentations.

### Risk Factors

The development of FG is strongly associated with several risk factors, most notably poorly controlled comorbidities that impair microvascular function. Key comorbidities include diabetes mellitus, alcohol use disorder, and immunocompromised states, such as those associated with HIV, malignancy, chemotherapy, or chronic steroids. Anatomic risk factors include urethral strictures, indwelling catheters, genital trauma, and perianal abscesses [5]. Most patients presenting with FG will possess multiple risk factors.

Diabetic patients are particularly susceptible to NSTIs, which is further complicated by the possible risk posed to patients by Sodium-Glucose Co-Transporter-2 inhibitors (SGLT2i). These medications work to lower serum glucose by inhibiting renal reuptake, thus causing glucosuria. This glucosuria, and the possibly increased risk of genital and perineal infections led to an FDA safety alert in 2018 [6]. However, subsequent high-level evidence has not demonstrated a significant difference in FG risk between SGLT2 inhibitors and other diabetes treatments [7]. The low incidence of FG may limit the ability to draw definitive conclusions at this time, and thus patients with scrotal infections should be considered for alternative diabetic medications.

### Mortality

FG carries a substantial mortality risk. While population-based data indicate a relatively stable overall mortality rate of approximately 7.5% in recent decades, single-center series often report higher rates, ranging from 20 to 40% [1, 8]. Inpatient-specific mortality has been reported between 4.7% and 7.3% [9, 10]. Established independent factors associated with mortality included advanced age and Medicaid insurance [9]. Sarcopenia has also been identified as a novel mortality risk factor [11]. The causes of mortality in FG are multifactorial, but generally involve sepsis leading to multiple organ failure [12]. Interestingly, high-volume centers and low-volume centers have similar mortality rates, despite more severe disease seen at high-volume centers [13], potentially suggesting that high-volume centers may have superior patient outcomes. Regardless, timely management is crucial and should not be delayed for patient transfer. Despite advances in the management of FG, longer-term morbidity and mortality data remain sparse, particularly regarding post-discharge survival.

### Severity Indices and Mortality Prediction

Given the high morbidity and mortality associated with FG, accurate assessment and prediction of patient outcomes are crucial. Several severity indices have been developed to aid clinicians in determining diagnosis and prognosis. The Fournier Gangrene Severity Index (FGSI) was initially proposed by Laor et al. in 1995 [14]. It incorporates nine clinical and laboratory parameters, each scored from 0 to 4, to assess disease severity. A total score above 9 indicates a 75% mortality probability, while a score at or below 9 suggests a 78% survival probability.

To enhance predictive accuracy, newer indices such as the Uludag Fournier Gangrene Severity Index (UFGSI) were developed. Introduced in 2010, UFGSI also incorporates age and disease extent, increasing mortality probability to 94% for scores above 9 and survival probability of 81% for scores at or below 9 [15]. The Simplified FGSI (SFGSI), focusing on fewer variables (potassium, creatinine, hematocrit only), offers a more user-friendly assessment while maintaining some predictive accuracy, particularly valuable in time-sensitive settings.

A recent systematic review of 40 studies (2,257 patients) found that non-survivors had higher FGSI, SFGSI, and UFGSI values at admission, with UFGSI demonstrating the highest predictive power (AUC: 0.94), followed by FGSI (AUC: 0.90) and SFGSI (AUC: 0.84) [16]. A subsequent analysis comparing FG-specific indices to disease-agnostic mortality indices found UFGSI and FGSI to be the most accurate, while non-specific indices like LRINEC were less reliable [17].

Other predictive markers, such as the C-reactive protein (CRP) ratio (preoperative CRP/postoperative CRP at 48 h), with higher values indicating a larger decrease in CRP, have been investigated [18]. Mean CRP ratios were reported to be higher in survivors (6.7 ± 6.6) compared to non-survivors (1.2 ± 0.8), and a cutoff value of 1.78 predicted death with high sensitivity (86%) and specificity (82%). These findings suggest that even simplified and clinically useful bedside tools have utility in predicting mortality.

## Pathophysiology, Microbiology, Diagnosis

### Pathophysiology

While often overlooked, considering the pathophysiology of FG is essential to understanding modern debridement techniques. The pathophysiology is centered on the rapid subcutaneous and fascial spread of a polymicrobial infection. Following disruption of tissue integrity via cutaneous injury or violation of the gastrointestinal or genitourinary tract, it is hypothesized that microbes seed the subcutaneous tissue and fascia. In high-risk patients with compromised microvasculature, tissue necrosis ensues, fueling the proliferation of pathogens [19, 20]. Infectious spread continues along the deeper fascial planes, compromising neurovascular structures; however, due to the rich and redundant vasculature within the skin, cutaneous manifestations may present much later than deep tissue necrosis [21]. This suggests that unless skin is overtly necrotic, it may still be salvageable even if the subcutaneous and fascial tissue below is compromised. This nuanced understanding of the spread of NSTIs is the foundation of skin-sparing debridement [22]. Skin-sparing techniques have become increasingly emphasized in modern treatment protocols, as preserving healthy tissue while removing only necrotic areas is crucial for maintaining reconstructive options.

### Microbiology

FG is associated with diverse microbial species due to the varied etiologies affecting the genitourinary region. In this context, FG may be considered a conglomerate endpoint of multiple disease processes, each with distinct microbiological profiles depending on the infection source. Compared to lower extremity NSTIs, FG more frequently involves gram-negative organisms; however, no predominant organism can be identified in approximately 25% of cases [23, 24]. FG can still be monomicrobial (30.1%), but the majority are polymicrobial infections (58.4%), with facultative and obligate anaerobes thriving in the hypoxic environment [25]. Common causative bacteria include *Escherichia coli*,* Klebsiella pneumoniae*,* Bacteroides fragilis*,* Streptococcus* species, *Pseudomonas* species, and *Staphylococcus* species. Recent studies identify *E. coli* as the most frequently isolated pathogen, followed by *Enterococcus faecium*,* Staphylococcus aureus*,* and Pseudomonas aeruginosa*. Less common isolates, such as *Acinetobacter* and *methicillin-resistant Staphylococcus aureus* (MRSA) can been associated with higher mortality rates [26].

### Diagnosis

While a diagnosis of FG can almost always be made clinically, advancements in imaging technologies have significantly enhanced early detection of the disease process, which is crucial to prevent rapid deterioration, widespread tissue loss, and death. However, while imaging aids diagnosis and surgical planning, especially when the diagnosis is uncertain, the decision to proceed with surgical intervention remains a clinical judgment.

To aide in diagnosis when uncertain, urologists can consider a variety of imaging modalities. Computed Tomography (CT) is invaluable in the diagnosis and surgical planning for FG and is considered the gold standard. The ability of CT to offer rapid, detailed, and comprehensive evaluation, including the assessment of gas within the soft tissues, makes it the preferred imaging modality. Recent studies have demonstrated the high accuracy of contrast-enhanced CT, with 100% sensitivity and 98% specificity [27]. CT also helps evaluate infection location and depth, guiding skin-sparing debridement and determining the need for additional surgical teams such as acute care surgery in widespread infections. Point-of-care ultrasound enables real-time, bedside assessment of abscesses, gas formation, and fluid collections, although this is particularly operator dependent. This imaging modality is particularly useful in the emergency department setting and may help make rapid decisions about surgical debridement [28, 29]. Magnetic Resonance Imaging (MRI) typically provides superior soft tissue analysis, making it theoretically useful in assessing the full extent of disease, especially in more complex cases. However, MRI’s slow acquisition time limits its practical role in emergency settings where rapid decision-making is necessary [30].

## Medical Management

### Antibiotics

Upon arrival to the emergency department, immediate empiric, broad-spectrum antibiotic therapy targeting polymicrobial infections is crucial in those with suspected FG. Initial regimens should include broad coverage for gram positive, gram negative, and anaerobic bacteria until the culprit(s) are identified via tissue culture. Therapy should then be tailored to culture results. Early collaboration with infectious disease specialists can be considered for optimal management when available. While local antibiograms and culture results should guide therapy [31], guideline recommended empiric regimens should be considered (Table 1) [32].

**Table 1:** Author recommendations for empiric antibiotics for Fournier’s gangrene based on IDSA guidelines. Recommended antibiotic regimens should be adjusted based on culture results

	Antibiotic Regimen	Coverage
Option #1	Linezolid + Piperacillin/Tazobactam	Linezolid targets gram-positives including MRSA with anti-toxin activity. Piperacillin/Tazobactam targets broad gram-negatives (aerobic and anaerobic).
Option #2	Vancomycin + Clindamycin* + Piperacillin/Tazobactam	Vancomycin targets gram-positives including MRSA. Clindamycin targets some gram-positives and anaerobic bacteria with anti-toxin activity. Piperacillin/Tazobactam targets broad gram-negatives (aerobic and anaerobic).
Option #3 (Penicillin Allergy Considerations)	Linezolid + Meropenem or Linezolid + Cefepime or Ceftriaxone + Metronidazole	Linezolid targets gram-positives including MRSA with anti-toxin activity. Meropenem targets broad gram-negatives (aerobic and anaerobic). Cefepime/Ceftriaxone targets broad gram-negatives (aerobic) while Metronidazole targets anaerobic bacteria.

MRSA - Methicillin-resistant Staphylococcus aureus

*Clindamycin is recommended for treatment of documented group A streptococcal necrotizing fasciitisand and can be considered while awaiting culture results

### Adjuvant Therapies

Several adjuvant therapies have been explored to attempt to improve outcomes in FG; however, most have not made it to routine practice. These therapies aim to address specific aspects of the disease process, such as enhancing tissue oxygenation, neutralizing bacterial toxins, or modulating the immune response. Their efficacy remains the subject of ongoing research, and their use should be carefully considered on a case-by-case basis at high-volume centers.

Hyperbaric oxygen therapy (HBOT) involves breathing 100% oxygen in a pressurized chamber, increasing plasma oxygen levels. This results in improved tissue oxygenation, enhanced leukocyte function, promotion of angiogenesis, collagen synthesis, and accelerated wound healing [33, 34]. While HBOT has been used to help control infection, reduce tissue edema, and potentially improve outcomes, its clinical efficacy remains controversial. Some studies suggest improved mortality outcomes, while others report no significant benefits [35–37]. Larger randomized trials are needed to determine its definitive role in FG. Furthermore, the practicality of offering HBOT for patients admitted with large wounds remains a challenge.

Clindamycin and Linezolid are often utilized to inhibit bacterial toxin production [38]. Similarly, Intravenous immunoglobulin (IVIG) has been studied as a potential adjunctive therapy, with the ability to neutralize a broader spectrum of bacterial toxins. While some evidence supports this potential benefit specifically in invasive group A streptococcal (iGAS) infections [39–41], other studies including a broader spectrum of pathogens commonly observed in FG, have not demonstrated a significant difference in mortality [42]. At this time the use of IVIG is not recommended.

Investigational immunomodulators such as Reltecimod were designed to mitigate organ dysfunction in critical illness by inhibiting T-cell stimulation. A 2020 trial showed promising trends in improved organ function, reduced mortality, and fewer NSTI debridements; however, no significant benefit was observed in the modified intent-to-treat analysis. Reltecimod remains unapproved by the FDA [43]. Targeted therapies that reduce organ dysfunction warrant further investigation, though no subsequent studies on CD28 T-lymphocyte receptor mimetics, or similar agents, have been published [44].

## Surgical Management of Fournier’s Gangrene

Modern surgical treatment of FG prioritizes prompt debridement with an emphasis on preserving viable skin tissue. This is unlike historical radical approaches which often resulted in the removal of excessive recoverable tissue, limiting future reconstructive options. The modern standard of care emphasizes excising only dead or severely compromised tissue rather than continuing to perfectly healthy, non-cellulitic tissue. Infected but viable tissue is then given a trial of salvage with aggressive resuscitation and antibiotic therapy. Multiple surgical debridements should be the norm while using this skin-sparing approach, with studies reporting a median of 1.8 to 3.5 debridements, and many requiring more [3, 45, 46].

### Skin-Sparing Debridement

Skin-sparing debridement is becoming a more commonly utilized surgical strategy for FG. In concordance with the pathophysiology, histological evidence supports the theory of skin-sparing debridement. One study of debrided tissue demonstrated that intact epidermal layers were intact in areas where the underlying subcutaneous tissue was necrotic [22], suggesting that skin without visible necrosis can be preserved. Further, even if ischemia subsequently develops, it can be removed at subsequent procedures. Differentiating necrosis, which requires immediate removal, from infected tissue that may recover with time was a key conceptual advance in FG.

Recent descriptions of strategic incisions to access deep tissues without compromising salvageable skin have led to the development of skin-sparing approaches, gaining popularity at high-volume centers. This strategy offers comparable outcomes in terms of length of stay and mortality, but with vastly improved wound and reconstruction outcomes when compared to traditional, more aggressive debridement methods [47–49]. One retrospective review of 487 patients by Tom et al. comparing traditional vs. skin-sparing debridement revealed comparable mortality rates with a significantly increased rate of primary closure (i.e. delayed primary closure or local tissue flaps) and reduced the need for complex reconstructive closures such as skin grafts [50]. This group noted a jump from 0% primary closure rate in the 230 traditional debridement patients to 50% in the 257 skin-sparing patients.

Optimal patient positioning during debridement is crucial for effective access and visualization. For most FG cases, lithotomy positioning provides wide exposure of the perineum, scrotum, anus and genitals. A Foley catheter should be placed to help identify the urethra. Suprapubic catheters are generally avoided unless there is direct urethral involvement, such as erosion or fistula.

**Fig. 1:**
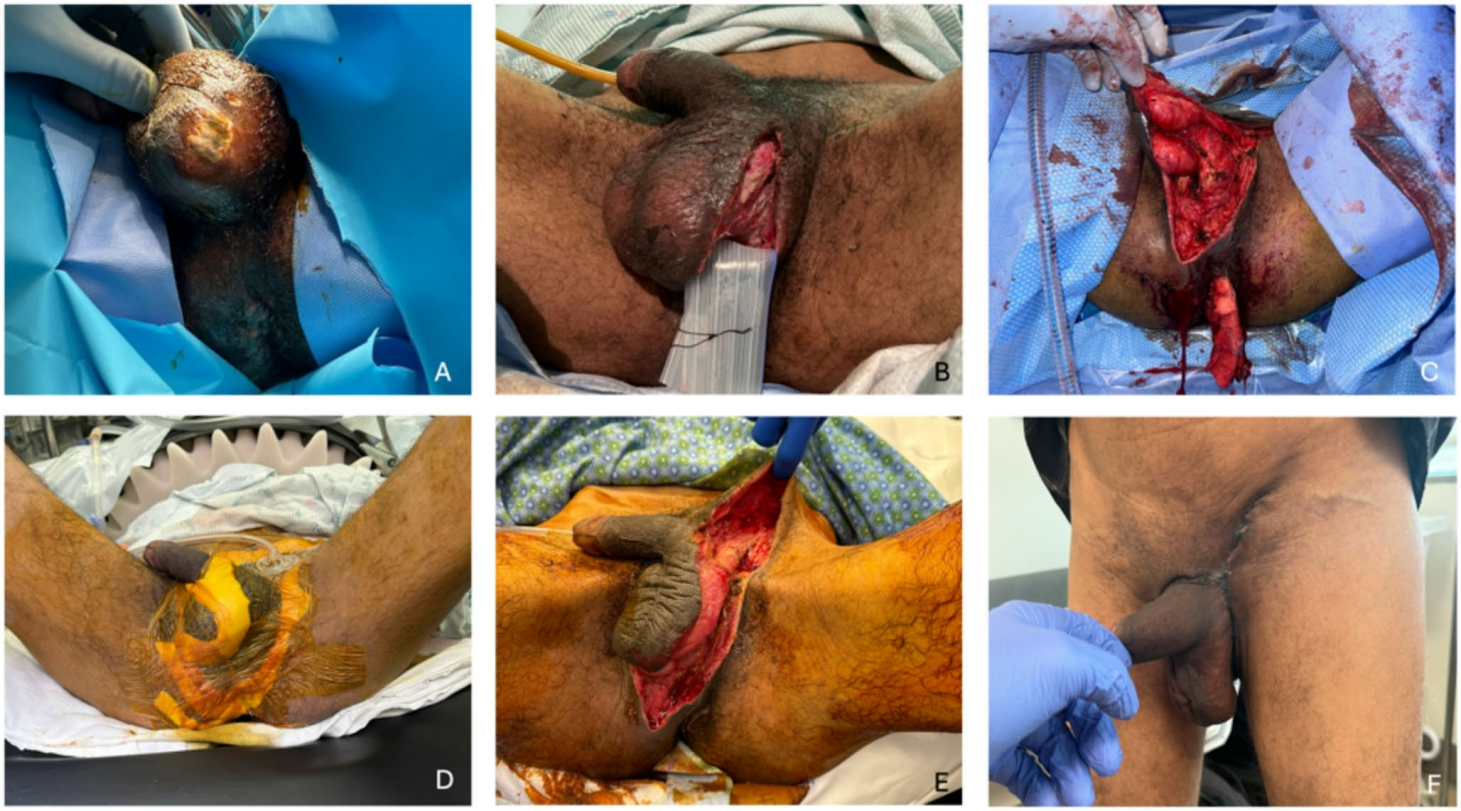
Surgical Management of Fournier’s Gangrene via skin-sparing technique and primary reconstruction. (**A**) Initial presentation showing necrotic skin prior to debridement. (**B**) Limited debridement with small incisions performed at the outside hospital. (**C**) Subsequent debridement at tertiary center with extension of incisions to improve surgical exposure. (**D**) Vacuum-Assisted Closure therapy applied between debridement procedures. (**E**) Final wound closure post skin-sparing debridement. (**F**) Follow-up at one month

Strategic incisions are essential to widely access deep tissues while preserving viable skin. One recommended approach is a long unilateral incision, approximately 2 cm medial to the inguinal crease [51]. This incision can extend to the ipsilateral anterior superior iliac spine and the skin overlying the gluteal muscles, offering ample exposure for debridement and scrotal/perineal reflection (Fig. 1). While some authors argue for a midline scrotal approach, this creates two skin flaps which both require retraction for tissue visualization as opposed to one which is ergonomically easier to manage intraoperatively. Once dissected down to facia, blunt finger dissection is often used to identify and separate healthy from necrotic tissue. A key distinction in skin sparing is tissue incision vs. excision. Incisions should be extended liberally to access and visualize necrotic tissue requiring excision, but excision of healthy tissue just for wound access is unnecessary in most cases. Once necrotic tissues are excised, the wound should be reexamined in the operating room in the coming days. Areas that initially appear viable may become ischemic due to tissue mobilization within 24–72 h, requiring reassessment and subsequent debridement. Untreated ischemic tissue may serve as a nidus for continued infection.

Adoption of skin-sparing techniques improves wound closure rates and reduces the need for subsequent complex reconstructive procedures [49]. This patient-centered approach aims to decrease the need for burdensome wound care and increase discharge to home rates, normalizing the patient earlier in their recovery. However long-term and patient-reported outcomes using this strategy warrant further study. Nonetheless, this technique facilitates highly functional wound closures without the need for complex reconstruction.

### Post-Debridement Wound Care

Skin-sparing techniques can add nuance and complexity to the patient’s wound care following the first debridement, especially when preserving infected but viable tissue. Therefore, high-quality wound care is critical to promote tissue salvage and minimize the risk of disease progression. In the early phases of post-debridement care, antimicrobial solutions are commonly used. For deeper, tracking wounds for which repacking might be insufficient, difficult, or painful for the patient, vacuum-assisted closure (VAC) dressings can be employed earlier in the treatment process [51].

### Antimicrobial Solutions

Dakin’s solution, first introduced in 1915 for battlefield wound care, continues to play an important role in post debridement wound dressings [52, 53]. Dakin’s active ingredient, dilute sodium hypochlorite, is bactericidal, and has been shown to stimulate fibroblast recruitment and wound healing. Although there are concerns regarding tissue cytotoxicity, studies have demonstrated that dilute concentrations retain bactericidal effects while minimizing harm to native cells [54]. Alternative adjunctive dressing solutions like povidone-iodine and chlorhexidine irrigation solutions have been used with limited comparative evidence. Other recent hypochlorous acid-based solutions (e.g., Vashe^®^ and PhaseOne^®^) have been developed and are effective against a wide range of bacterial biofilms, sterilizing most biofilms within 10 min [55]. These newer solutions may provide a promising alternative to Dakins, but further investigation is needed to determine and compare efficacy.

### Negative Pressure Wound Therapy

Negative pressure wound therapy (NPWT), commonly known as VAC therapy, remains a valuable tool in managing FG wounds. While the complex genitourinary anatomy can pose challenges to maintaining a proper vacuum seal, NPWT dressings offer several advantages over conventional dressings, including minimizing uncomfortable dressing changes, reducing pain, increasing mobility, and optimizing wounds for reconstruction [45, 56, 57]. Although variations in vacuum sealing techniques exist, the underlying principle remains consistent: active suction of the wound bed removes exudative fluid and stimulates tissue repair. However, the impact of NPWT on key clinical outcomes compared to traditional dressings remains undetermined. While some studies demonstrate clinical benefit, others suggest no significant impact on mortality or disease severity [58, 59]. Nonetheless, while the evidence for NPWT is equivocal and low-quality, NPWT remains a standard treatment for FG wounds in our practice due to their complexity and large surface area, making adequate standard dressing changes a challenge. Future research on NPWT in FG should explore its association with survivorship outcomes like time to wound closure, rate of wound closure, time to discharge and discharge status as opposed to mortality, which is less likely to see an impact from variations in wound care strategies.

### Reconstructive Strategies

Following initial debridement, various reconstructive strategies can be employed to facilitate wound closure, especially in cases in which skin-sparing debridement was not performed. For shallow, limited defects where minimal intervention is desired, secondary intention healing can be considered, however, primary closure is preferred. In cases with significant tissue loss, loco-regional flaps, such as fasciocutaneous or myocutaneous flaps, become necessary, providing robust blood supply for optimal tissue coverage. Skin grafting serves as another option when flaps are not adequate or feasible. A recent systematic review of 38 studies involving 576 FG reconstructions revealed that simpler techniques, including direct closure, secondary healing, skin grafts, and local flaps, were used in the majority of reconstructions (77.6%). More complex loco-regional flaps accounted for 22.4% of cases, with no reports of free flaps [60]. The choice of reconstructive strategy ultimately depends on the extent of tissue loss, wound location, and surgical expertise, as well as the individualized goals of the patient. This final point is essential as many of these patients are older, debilitated from chronic and critical illness and may do better with simpler closures even if they are less cosmetic.

Healing by secondary intention, involving granulation and epithelialization, is best suited for shallow, limited defects [61]. Although secondary intention was historically commonplace, modern techniques emphasize primary closure by urologists to minimize wound care needs and expedite healing and recovery. Ideally, delayed primary closure is preferred, with one recent series reporting a median of 2.5 debridements and 6.5 days prior to closure, and average length of stay of 13 days [62] in patients closed primarily. Hospital length of stay has been reported to be a week less on average for those patients closed primarily compared to those with wounds left to heal via secondary intent, with comparable infectious outcomes [63]. A 2022 study by Sandberg et al. reported that primary closure can decrease wound convalescence time by over 60%, even when adjusting for wound size and critical illness [64]. For small scrotal defects, simple closure of remaining scrotal tissue yields good cosmetic results, especially when ≤ 50% tissue is removed; however, advanced scrotal closure techniques have been widely reported for various disease severities [65]. While closure may superficially seem to be a source of longer hospital stays and increased cost, preliminary data from high-volume institutions have demonstrated that when outpatient wound care and the time required to arrange outpatient services and discharge are accounted for, delayed primary closure may be more cost effective [66].

Local and regional flaps transfer tissue with its blood supply from a nearby location. They are advantageous for small to moderate defects and provide well-vascularized tissue. These include random flaps (40.4%) and flaps based on known vascular anatomy (59.6%) [60]. The genitourinary region’s rich vascular supply and tissue elasticity provide multiple advanced reconstructive options including anterolateral thigh, superomedial thigh, groin, and SCIP flaps [65, 67–69]. These procedures are often reserved for well-selected cases and performed in specialized centers [60, 68]. The Limberg flap is a rhombus-shaped local random-pattern transposition flap, meaning it is designed from adjacent skin and rotated into the defect to achieve tension-free closure [70]. This flap is less complex and can be easily adopted into most surgeons’ armamentarium. Myocutaneous flaps, such as gracilis flaps, include muscle tissue along with skin and subcutaneous layers, and are typically reserved for larger, deeper defects and provide greater durability after extensive tissue loss. When more complex flaps are being considered, most urologists will enlist the assistance of a plastic surgeon.

For larger defects, skin grafting also offers a versatile reconstructive option [69, 71]. Split-thickness grafts, harvesting a thin layer of skin, are commonly used, while full-thickness grafts, including all skin layers, are uncommon in FG as full-thickness harvest sites require subsequent closure. Split-thickness skin grafts are generally preferred for their improved take, but full-thickness grafts may offer more functional skin in the genital region [71]. Skin grafts are effective for closure, but graft contraction can be a bothersome complication.

In cases of severe tissue loss, adjunctive maneuvers such as testicular transposition into the subcutaneous tissues of the thighs (thigh pouches) or tissue expanders may be explored. Testicular transposition involves relocating the testicles and spermatic cords to a pouch in the upper thigh, often utilized for extensive scrotal defects. While relatively simple with few complications, this procedure is often not offered due to concerns regarding potential negative impacts on testicular function, temperature regulation, and reported cases of testicular atrophy and pain [69, 72, 73]. However, in our experience, many patients prefer the simplicity and quicker recovery of thigh pouches to the more complex wound care needed for testicular coverage like skin grafts or flaps. In our practice, we offer thigh pouches to assist with upfront closure, especially for frail, debilitated patients, and then offer elective reconstruction of the scrotum in follow-up. Anecdotally, many patients elect to defer further surgery and keep thigh pouches, even with regular follow-up. Tissue expanders can be considered to stretch remaining skin but are contraindicated in cases of complete scrotal skin loss [65].

While reconstructive techniques can improve functional and aesthetic outcomes, it is important to note that patient-reported outcomes in the context of FG are still poorly understood. To date, no studies have specifically employed patient-reported outcome measures after genital reconstruction. However, reconstructions that allow for primary closure and timely wound management save significant costs, benefiting both the healthcare system and presumably patients’ quality of life. As reconstructive techniques continue to evolve, future studies exploring the long-term outcomes and patient satisfaction with different reconstruction methods will be crucial in refining treatment protocols.

## Conclusion and Future Directions

This review provides a comprehensive overview of the contemporary management of Fournier’s Gangrene, optimizing survivorship, with an emphasis on the patient-centered paradigm shift towards skin-sparing debridement and genital reconstruction. Early recognition and prompt medical and surgical intervention remain mainstays of FG management, allowing for tissue salvage via skin-sparing strategies. The modern management of FG aims to improve functional outcomes earlier in a patient’s long recovery via early closure when feasible. However, there are several gaps in the literature that could help further optimize our care for these patients. Nearly 50% of FG patients seek medical care for similar symptoms prior to their NSTI diagnosis, emphasizing the importance of further research on early identification and prodromal FG [74]. Additionally, little is known about FG patients after their index admission, warranting further research on longer term post-hospital outcomes and patient reported outcomes after reconstruction. Continued research on subjective and objective outcomes after FG reconstruction is crucial to further refine treatment protocols and ultimately improve the quality of life for individuals affected by this devastating condition.

## Key References

*4. Abbasi B, Hacker E, Ghaffar U, Hakam N, Li KD, Alazzawi S, et al. Higher morbidity and mortality in females with fournier gangrene compared with males: insights from national inpatient sample data. J Urol. 2025;213(1):99-109.
  - New insights on mobidity and mortality.
**22. Alyanak A, Cakici OU, Turkmen YA. Skin-sparing approach in the management of Fournier's gangrene: the initial histological evidence and results of a tertiary health-care center. ANZ J Surg. 2022;92(1-2):128-31.
  - Pathophysiology support for skin sparing.
*50. Tom LK, Maine RG, Wang CS, Parent BA, Bulger EM, Keys KA. Comparison of Traditional and Skin-Sparing Approaches for Surgical Treatment of Necrotizing Soft-Tissue Infections. Surg Infect (Larchmt). 2020;21(4):363-9.
  - Outcomes after skin sparing technique.
**51. Koch GE, Amighi A, Skokan AJ, Hagedorn JC. Skin-sparing debridement in fournier's gangrene. J Urol. 2024;212(6):912-4.
  - Surgical insights and tecniques of skin sparing debridement.
*60. Susini P, Marcaccini G, Efica J, Giuffrè MT, Mazzotta R, Caneschi C, et al. Fournier's gangrene surgical reconstruction: A Systematic Review. J Clin Med. 2024;13(14).
  - Recent evidence on reconstruction in Fournier’s Gangrene.

## Data Availability

No datasets were generated or analysed during the current study.
